# Visible Light Induced Photochemistry of IO_2_^**–**^

**DOI:** 10.1021/acs.jpclett.6c01153

**Published:** 2026-05-28

**Authors:** Catherine S. Kellow, Jacob J. Newman, Jemma A. Gibbard

**Affiliations:** Department of Chemistry, 3057 Durham University, Durham DH1 3LE, United Kingdom

## Abstract

IO_2_^–^ and IO_2_ have been detected in the atmosphere and suggested as reactive intermediates in the destruction of ozone. Here, photoelectron action spectroscopy provides evidence for a vibrational progression of anion resonances, characterized by a vibrational frequency of ∼0.03 eV, originating from a weakly bound (close to the photodetachment threshold) electronically excited state of IO_2_^–^. Photoelectron spectroscopy of IO_2_^–^ at visible wavelengths shows indirect electron loss processes, likely autodetachment of the vibrational resonances leading to internally excited IO_2_, alongside direct detachment. Given that the excited state has a vertical excitation energy in the visible portion of the solar spectrum, it is likely that photoexcitation of IO_2_^–^ is occurring in the atmosphere, providing a new route for the formation of electrons and internally excited IO_2_.

## Graphical abstract

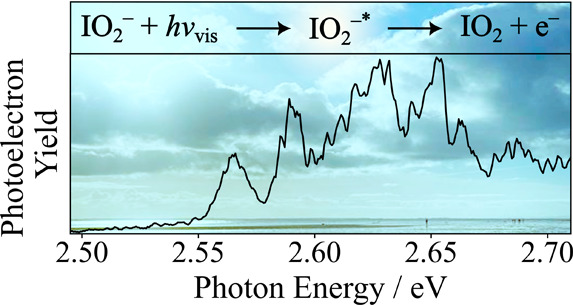

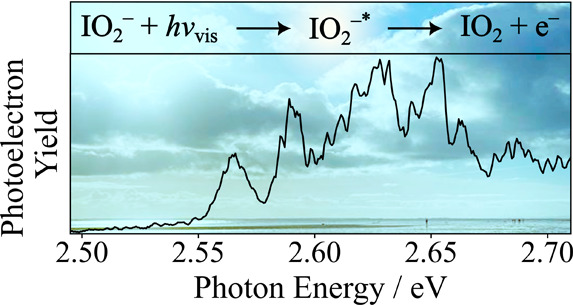

Atmospheric iodine levels have experienced a 3-fold increase in the last 75 years, according to ice core measurements.^1–3^ The reaction of oceanic iodide with atmospheric O_3_, produces iodine oxides, which are thought to be the largest source of gaseous iodine in the atmosphere.^3^ While this leads to ozone depletion, in conjunction with the better studied chemistry of anthropogenic chlorine and bromine containing species, the iodine oxides also readily undergo photochemistry which leads to the presence of many other iodine-containing species in the atmosphere.^3–6^ Much of the interest in this chemistry stems from the discovery that iodine oxides and their acidic counterparts can nucleate new particle formation, at rates which compete with the well-known sulfuric acid-ammonia particle formation pathway in certain coastal regions.^4,7–9^ Several studies have suggested that IO_2_ and IO_2_^–^ are intermediates in the stepwise depletion of O_3_, either by the reaction of iodide in seawater or iodine radicals produced by photolysis in the atmosphere, and furthermore, IO_2_ may be a source of particle nucleation events.^3,7,10–12^ Both IO_2_ and IO_2_^–^ have been directly observed in the atmosphere.^13,14^ Given the atmospheric significance of the iodine oxides, and IO_2_/IO_2_^–^ in particular, it is important that laboratory studies are undertaken to investigate the structure and photochemistry of these molecules, as such fundamental data are needed for the inclusion of iodine chemistry in climate models.^15^

Ion spectroscopy has been used to investigate anionic and neutral iodine oxides of atmospheric relevance to date, ranging from the simplest IO^–^ to much larger clusters, such as (I_2_O_5_)_3_(IO_3_^–^).^4,16–19^ The first photoelectron spectroscopy study of IO_2_^–^ (bent OIO^–^) was published over 30 years ago by Gilles et al., and reported an adiabatic detachment energy (ADE) of 2.577 ± 0.008 eV.^17^ Upon photodetachment, a well-resolved vibrational progression was observed up to *v*_1_ = 3, which was attributed to excitation of the symmetric stretch (ν_1_ = 765 ± 25 cm^–1^) in the neutral electronic ground state, with some excitation of the bending mode also seen (ν_2_ = 192 ± 35 cm^–1^).^17^ A hot band in the spectrum allowed the frequency of the symmetric stretch of the anion to also be reported (ν_1_ = 675 ± 25 cm^–1^).^17,20^ A recent Franck–Condon simulation study of IO_2_^–^ from Xu validated these assignments.^21^ Wen et al. also studied IO_2_^–^ with cryogenic photoelectron spectroscopy at higher photon energies (*hν*) and electronic structure calculations, which confirmed the presence of a hot band.^16^ From this, the energies of the neutral excited states from the A band (ADE = 4.20 ± 0.10 eV) up to the F band (ADE ∼ 7.8 eV) were reported, and their computations suggested that both IO_2_^–^ and IO_2_ are bent (C_2v_) molecules.^16^ While the spin–orbit splitting (ΔE_SO_) of I (ΔE_SO_ = 0.94 eV) and IO (ΔE_SO_ = 0.26 eV) are large, computations indicate that the contribution is much smaller for IO_2_ (ΔE_SO_ = 0.01 eV), which is consistent with the previous photoelectron spectroscopy studies of IO_2_^–^.^17,18,21–23^

Despite the previous ion spectroscopy studies, questions remain about the structure and dynamics of IO_2_^–^/IO_2_. First, it is unclear whether the anion has excited states, which may be key to understanding the photochemistry of IO_2_^–^. Second, though calculations have predicted the electronic structure of IO_2_^–^ and experiments have assigned the spectral features, a direct, experimental determination of the nature of its high-lying molecular orbitals has not been undertaken.^17,21^ To answer these questions, we have studied IO_2_^–^ using photoelectron imaging and photoelectron action spectroscopy at *hν* close to the photodetachment threshold i.e. near the ADE of IO_2_^–^. Our measurements have determined the symmetry and electron occupancy of the highest occupied molecular orbital of IO_2_^–^(^1^A_1_) to be (b_1_)^2^ and provided evidence for the presence of a weakly bound electronically excited anion state (IO_2_^–^(^1^B_1_)), which gives rise to a vibrational progression of autodetaching resonances.

Photoelectron images and action spectra were recorded on a photoelectron imaging spectrometer, which has been previously described in detail.^24,25^ Briefly, IO_2_^–^ was produced using electrospray ionization of a solution of NaIO_3_ in methanol. The anions entered the apparatus via a capillary, before being guided using radiofrequency ion guides and accumulated in an ion trap, thermalized by collisions with room temperature He. Controlled collision-induced dissociation in the ion guides produced IO_2_^–^ from the IO_3_^–^ parent, before the ions were accelerated and mass-selected using a Wiley–McLaren time-of-flight mass spectrometer.^26,27^ Photoelectrons were produced through the intersection of nanosecond laser pulses (Nd:YAG pumped optical parametric oscillator) of variable *hν* and mass-selected ion packets of IO_2_^–^. The resulting photoelectrons were imaged on a position sensitive detector, arranged at the focus of a velocity map imaging setup.^28^ The photoelectron images were processed using the maximum entropy Legendre expanded image reconstruction (MELEXIR) algorithm, to extract electron kinetic energy (eKE) spectra, which have a three-point rolling average applied, and photoelectron angular distributions (PAD).^29^ PADs are characterized by an anisotropy parameter, – 1 < β_2_ < 2, which provides symmetry information about the molecular orbital from which the electron is photodetached.^30–32^ The energy resolution of the photoelectron images is $\frac{\Delta E}{E}=5\%$ , as extracted from the well-known photoelectron spectrum of I^–^.^23^

Photoelectron action spectroscopy, where the total yield of photoelectrons is recorded as a function of *hν*, provides an insight into the location of excited states and can function as a gas-phase analogue to absorption spectroscopy.^33–36^ The photoelectron action spectrum was normalized for photon flux, assuming that all electron loss processes are one-photon, to account for the fluctuations in the laser power with *hν* and had a three-point rolling average applied.

Ground and excited state electronic structure calculations were performed on IO_2_ and IO_2_^–^. Given the large number of electrons in IO_2_^–^/IO_2_ and the potential for a dense manifold of electronic states, it is expected that the computational results will provide a qualitative picture of the electronic structure only, in order to aid the interpretation of the experimental data. Ground state geometry optimizations and electronic structure calculations were performed using the Gaussian 16 package, at the B3LYP level of theory with the aug-cc-pVTZ-pp basis set (using pseudopotentials for the iodine atom).^37–41^ This level of theory has previously been used to study the electronic structure of IO_2_^–^ and IO_2_ and our computed ground state geometries and energetics were in excellent agreement with the previously reported computations of Wen et al.^16,40^ Furthermore, we extended this previous work by performing excited state calculations on IO_2_^–^ (Gaussian 16/B3LYP/aug-cc-pVTZ-pp) using the Tamm–Dancoff approximation and natural transition orbital analysis.^42,43^

Photoelectron spectra of IO_2_^–^ were recorded at *hν* = 2.58 – 4.07 eV, but as similar spectral features were observed at many different *hν*, only a selection are shown in Figure 1. The spectra are reported on eKE and electron binding energy (eBE + eKE = *hν*) scales and have been normalized to the most intense spectral feature. The spectra show two distinct features. The first is a series of equally spaced peaks at constant eBE ∼ 2.50 eV, 2.58 eV, 2.67 eV, 2.77 eV, and 2.87 eV, which are partially present at all *hν* studied, and are highlighted in red in Figure 1. The second feature is a series of additional peaks at low eKE, which appear to shift on an eBE scale, and are only present in the photoelectron spectra recorded between *hν* = 2.56 – 2.74 eV. These peaks are highlighted in blue in Figure 1.

**Figure 1:**
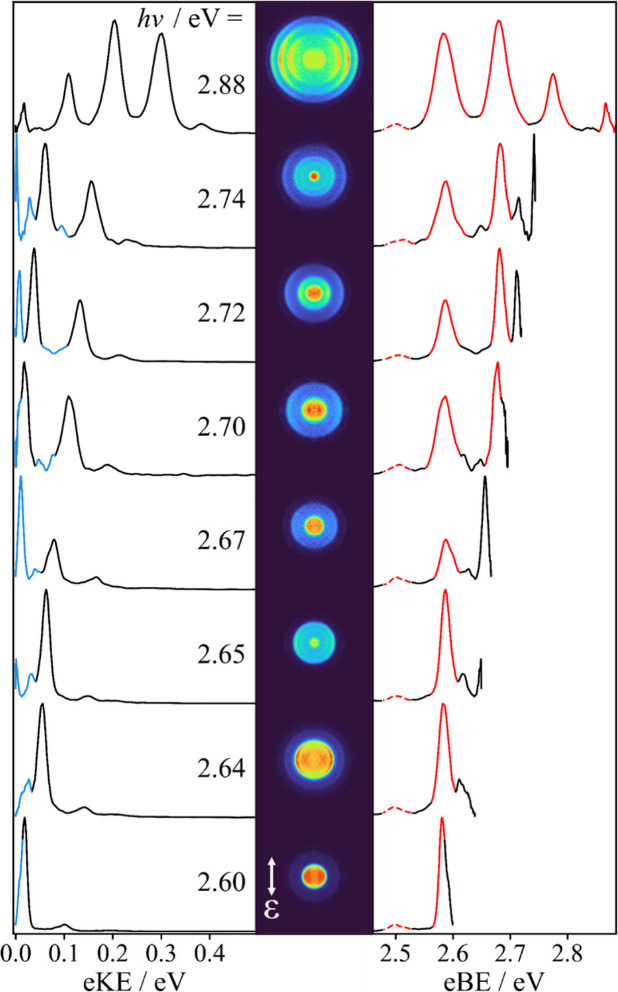
Photoelectron spectra of IO_2_^–^ recorded at selected *hν* and reported on an eKE (left) and eBE (right) scale, alongside the corresponding photoelectron images (center). The indirect electron loss channel is highlighted in blue (left-hand side, eKE scale), and the direct detachment channel is shown in red (right-hand side, eBE scale), with the hot band indicated by a red dashed line (right-hand side, eBE scale).

The fixed eBE feature (red, Figure 1), as typified by the *hν* = 2.88 eV IO_2_^–^ photoelectron spectrum, has been observed in the previous photoelectron spectroscopy studies of IO_2_^–^ reported by Gilles et al. and Wen et al. at shorter wavelengths.^16,17^ As described in the introduction, this fixed eBE feature arises from direct detachment of IO_2_^–^ (red lines, Figure 1) and a hot band (red dashed line, Figure 1).^16,17^ From this feature we report an experimental ADE ∼ 2.58 eV in excellent agreement with the previous higher resolution studies.^16,17^ The relative population of the hot band compared to the direct detachment band is ∼4%, as determined by integrating the associated spectral features (i.e., red dashed vs red in Figure 1). This is consistent with an ion temperature of T ∼ 300 K, where a Boltzmann distribution analysis would predict the population of the vibrational level to be

$$\frac{N_{\nu_{1}=1}}{N_{\nu_{1}=0}}∼e^{-h\nu_{1}/k_{B}T}∼4.5\%$$

in the anion, as ν_1_ = 0.08 eV.^17^ Finally, as we have performed photoelectron imaging on IO_2_^–^ for the first time, we have also gained information on the PADs. The spectral features of the direct detachment channel (*v*_1_ = 0, 1, 2, 3, red, Figure 1) have strongly anisotropic PADs, which for the photoelectron spectrum recorded at *hν* = 2.88 eV are characterized by β_2_ ≈ −0.9 ± 0.1, −0.9 ± 0.1, −0.8 ± 0.1 and −0.6 ± 0.1 for peaks with increasing eBE (*v*_1_ = 0 → 3). The PAD of the hot band (eBE ∼ 2.5 eV, red dashed, Figure 1) is characterized by β_2_ ≈ −0.8 ± 0.1. At lower *hν* all of the PADs are more isotropic as a result of Wigner’s law.^44^ It should be noted that while we are the first to perform photoelectron imaging on IO_2_^–^, Gilles et al. gained some preliminary information about the PADs by recording the photoelectron spectra using different laser polarizations, and assigned a β_2_ = – 0.71 for direct detachment to IO_2_ *v*_1_ = 1 at *hν* = 3.53 eV.^17^

In conjunction with Koopmans’ theorem, which predicts that photodetachment will result in electron loss from the highest occupied molecular orbital (HOMO) of the anion, we can use the PADs to understand the symmetry of the HOMO of IO_2_^–^.^30,31,45^ Therefore, β_2_ ≪ 0 indicates that the anion HOMO has significant *p* or π character.^30,31,45,46^

A molecular orbital diagram for IO_2_^–^ (C_2v_) is shown in Figure 2, constructed from the partially filled atomic orbitals of I and O e.g. 5p and 2p, and ordering the different molecular orbitals based on the likely extent of atomic orbital overlap.^16,17,47^ While this is a very simple approach, the relative ordering of the molecular orbitals shown in Figure 2 is consistent with the results of our electronic structure calculations. The molecular orbital diagram may be populated in a singlet (^1^A_1,_ (b_1_)^2^ shown in Figure 2) or triplet (^3^B_1_, (b_1_)^1^ (a_1_)^1^) configuration, such that, in accordance with Koopmans’ theorem, electron loss would be expected from the (b_1_) or (a_1_) orbital.^45^ As the HOMO of the ^1^A_1_ state is composed of contributions of I­(5p_*x*_) overlapped with O­(2p_*x*_), it likely has pure π character, and photodetachment from this orbital would lead to a strongly anisotropic PAD (β_2_ ≪ 0). In contrast, the HOMO of the ^3^B_1_ state is composed of contributions from I­(5p_*z*_) overlapped with O­(2p_*z*_) and O­(2p_*y*_), which, when considered in the bent geometry (IO_2_ sits in the *yz* plane), is likely to have mixed π and σ character, leading to a more isotropic PAD following photodetachment (β_2_ ∼ 0). Given that our observed experimental PAD has β_2_ ∼ −0.8, this picture supports the assignment that IO_2_^–^ has a (b_1_)^2^ electron configuration and therefore its ground state is ^1^A_1_. This result is replicated by our electronic structure calculations and recent theoretical work by Xu.^21^ Furthermore, our molecular orbital diagram (Figure 2) indicates that upon photodetachment, an electron is removed from an antibonding orbital, which may be expected to result in a shorter I–O bond in the neutral vs the anion. This is consistent with the observation of excitation of the symmetric stretch vibrational mode upon direct photodetachment (red, Figure 1).^17^

**Figure 2:**
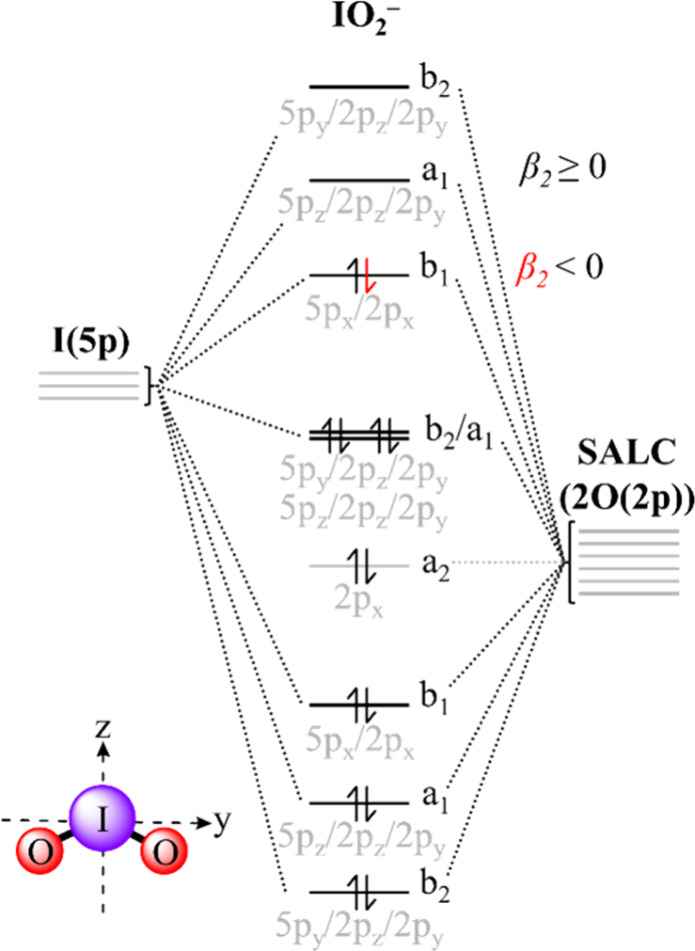
Molecular orbital diagram for IO_2_^–^. Photodetachment would be expected to remove the red electron and result in a PAD with negative anisotropy.

The second feature in the photoelectron spectra of IO_2_^–^, which is highlighted in blue in Figure 1, is a series of low eKE peaks at eKE ∼ 0.01 eV, 0.04 eV, 0.07 eV, and 0.09 eV, which are present in the photoelectron spectra recorded over a narrow range of *hν* = 2.56–2.74 eV only and have not been reported previously. While the peaks shift linearly on an eBE scale with changing *hν*, the peaks are fixed on an eKE scale. Furthermore, as the *hν* increases, the number of fixed eKE features observed in the photoelectron spectra increases. Taken together this indicates that we are observing indirect electron loss mediated via an electronically excited anion state, which is resonantly excited at *hν* = 2.56–2.74 eV.

The most plausible assignment for the indirect electron loss pathway is autodetachment, where excitation to an anion resonance, which is metastable with respect to detachment, leads to electron loss.^48,49^ The eKE of the ejected photoelectron is determined by the energy difference between the anion resonance and the neutral final state, and appears fixed on an eKE scale with changing *hν*.^48^ For the higher eKE spectral features (eKE ∼ 0.04 eV, 0.07 eV, and 0.09 eV), the assignment of autodetachment is conclusive, but for the lowest eKE peak (eKE ∼ 0.01 eV), we may be observing autodetachment from an electronically excited anion state, which is close in energy to the final neutral state, or thermionic emission, where a hot, ground state anion ‘boils off’ an electron.^50–52^ In the latter case, resonant photoexcitation to an electronically excited state of IO_2_^–^ would occur, followed by internal conversion (IC) to the ground state, forming a vibrationally hot anion which can lose an electron statistically. Given the observation of other autodetachment features in the photoelectron spectra though, and that the maximum intensity of the peak is eKE = 0.01 eV rather than eKE = 0.00 eV, it is most likely that we are observing autodetachment exclusively.

The PADs of the electrons lost via autodetachment (blue, Figure 1) have a weak negative anisotropy characterized by β_2_ ∼ −0.2 ± 0.2, where the larger error bars arise from the low relative intensity of the autodetachment spectral features compared to direct detachment. At similar eKE, i.e. near eKE ∼ 0 eV, the direct detachment peaks (red, Figure 1) are also characterized by a weak negative anisotropy, as a result of Wigner’s law.^44^ Therefore, for IO_2_^–^, the PADs do not help to distinguish between the direct and indirect electron loss pathways.

As we see clear evidence for indirect electron loss mediated by an excited electronic state of IO_2_^–^ in the photoelectron spectra, we performed photoelectron action spectroscopy in order to locate and identify the excited state. Figure 3 is the photoelectron action spectrum of IO_2_^–^, recorded between *hν* = 2.50–2.71 eV, and normalized to account for changes in photon flux with *hν,* assuming that all electron loss processes (direct or indirect) are one-photon. In this *hν* region, which is close to the threshold of photodetachment (near the ADE of IO_2_^–^), the photoelectron action spectrum of IO_2_^–^ consists of a series of equally spaced (∼0.03 eV) peaks in the photoelectron yield. The onset of electron loss is seen near *hν* ∼ 2.56 eV, which is slightly lower in energy than the ADE of IO_2_^–^ (ADE ∼ 2.58 eV, red, Figure 3) and the maximum photoelectron yield is seen at *hν* = 2.66 eV. Beyond *hν* = 2.71 eV, low laser power results in data with a poor signal-to-noise ratio, and so is not reported here.

**Figure 3:**
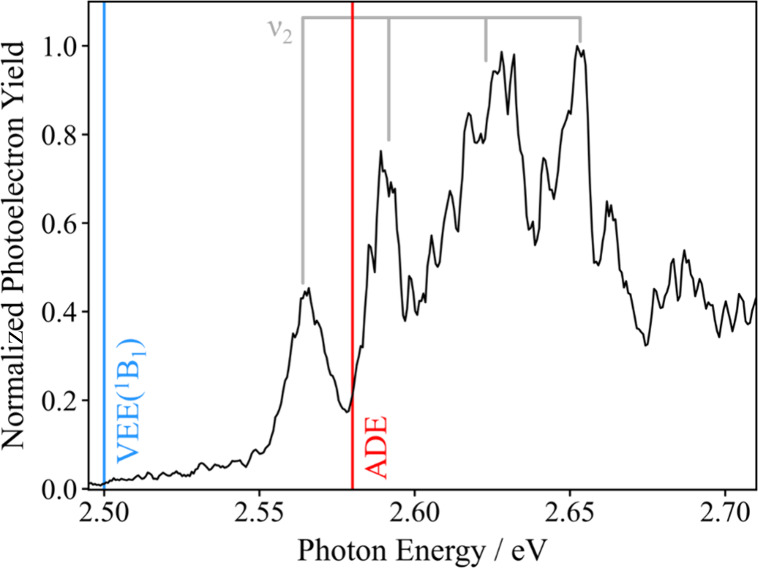
Photoelectron action spectrum of IO_2_^–^ (black, left axis) recorded between *hν* = 2.50 – 2.71 eV and normalized to account for changes in photon flux with *hν*. The experimental ADE (red) and the calculated VEE­(^1^B_1_) of IO_2_^–^ (blue) are denoted by vertical lines. Furthermore, a gray comb highlights the equally spaced (ν_2_ = 0.03 eV) peaks in the spectrum.

As direct detachment dominates the photoelectron spectra of IO_2_^–^ rather than indirect electron loss (Figure 1), changes in the direct photodetachment cross-section (σ) near threshold are expected to obey Wigner’s law, and therefore an unstructured photoelectron action spectrum is predicted.^44^ Specifically, as the angular momentum of the bound electron being detached is *l* = 1, the outgoing electron is expected to have s-wave and d-wave character, the latter of which is suppressed by the centrifugal barrier, and as such σ $\propto \;{\mathrm{eKE}}^{1/2}$ close to the photodetachment threshold. This picture likely describes the underlying structure of the photoelectron action spectrum, but not the peaks observed in Figure 3.^53,54^

Alternatively, electronically excited anion states above the photodetachment threshold, i.e. anion resonances, would be expected to result in sharp peaks in the photoelectron action spectra, as anion resonances are metastable with respect to electron loss and typically lead to autodetachment. However, as electron loss can only be mediated via an anion resonance following photoexcitation, the increased electron yield related to the anion resonance only occurs over a relatively narrow range of wavelengths (compared to photodetachment) where the photoexcitation cross-section is significant. Therefore, peaks in the photoelectron action spectra are likely to reflect the photoabsorption of electronically excited states of IO_2_^–^, assuming that photoexcitation leads to electron loss.

As the onset of electron loss is seen near *hν* ∼ 2.56 eV, which is slightly lower in energy than the ADE of IO_2_^–^ (ADE ∼ 2.58 eV, red, Figure 3), the first peak is attributable to a bound anion state, just below threshold (with higher energy peaks arising from unbound states i.e. resonances). The excited state electronic structure calculations also predict that the first excited state of IO_2_^–^(^1^B_1_) is weakly bound, with a computed vertical excitation energy VEE­(^1^B_1_) = 2.50 eV, which is shown on Figure 3 by a blue line. As the computed ADE = 2.57 eV of IO_2_^–^ is in excellent agreement with the experimental value, it indicates that the excited state may be lower in energy than the onset of electron loss in the action spectra. While resonant photoexcitation at *hν* = 2.56 eV does not provide enough energy for electron loss by itself (∼0.02 eV below ADE), it is likely that the internal excitation in the anions (anion *T* ∼ 300 K) can contribute, as evidenced by the observation of a hot band in the photoelectron spectra (dashed red line, Figure 1). However, if the electronic excited state were even lower in energy (i.e., VEE­(^1^B_1_) closer to 2.50 eV than 2.56 eV), then the thermal energy in the anions may be insufficient to lose electrons efficiently following photoexcitation at *hν* ∼ VEE.

As the series of peaks in the photoelectron action spectra is equally spaced (gray comb, Figure 3), with each peak ∼0.03 eV (∼240 cm^–1^) from its neighbor, the spectral structure suggests a vibrational progression. The associated frequency is different from the known vibrational frequencies of the anion (ν_1_ = 675 cm^–1^/0.08 eV) or neutral (ν_1_ = 765 cm^–1^/0.10 eV or ν_2_ = 192 cm^–1^/0.02 eV) and therefore is likely to be the vibrational frequency of a mode of the electronically excited anion state (^1^B_1_).^17^

Furthermore, electronic structure calculations indicate that the excited state arises from a pure HOMO to LUMO transition (^1^B_1_, > 99% (b_1_) → (a_1_)). From this we can speculate that photoexcitation would result in excitation of the bending mode. Largely this is due to the (b_1_) orbital having significant I­(5p_*x*_) character, while the (a_1_) orbital has I­(5p_*z*_) character, such that photoexcitation, i.e. promotion of an electron from (b_1_) to (a_1_) (Figure 2), is likely to result in a change of bond angle between the ^1^A_1_ and ^1^B_1_ state leading to excitation of the bending mode. This is supported by the observation that the magnitude of the excited vibrational frequency of ^1^B_1_ (ν_2_ = 0.03 eV) is much closer to the known energy of the bending mode of IO_2_ (ν_2_ = 0.02 eV), than the stretching mode of IO_2_ or IO_2_^–^ (ν_1_ = 0.1 eV, ν_1_ = 0.08 eV).^17^ To confirm this picture, we attempted geometry optimizations on the IO_2_^–^(^1^B_1_) state, but these would not converge, likely due the presence of a conical intersection between the ^1^A_1_ and ^1^B_1_ state at large bond angles.^200,201^ However, we were able to compute the energies of IO_2_^–^(^1^B_1_) as a function of bond angle and at fixed bond length, which showed a minimum near ∼ 155°, indicating that the minimum energy geometry of IO_2_^–^(^1^B_1_) likely has a larger bond angle than the IO_2_^–^(^1^A_1_) ground state.

Vibrational progressions in the photodetachment action spectra of anions have been observed before for dipole bound anion states, and with a computed dipole moment of 3.8 D, IO_2_^–^ is likely to have a dipole bound state.^55^ However, the electronic structure calculations predict that photoexcitation produces the valence bound anion state, IO_2_^–^(^1^B_1_). This is supported by the observation that the vibrational progression in the electronically excited anion state (ν_2_ ∼ 0.03 eV, gray comb, Figure 3) has a different frequency to the known bending mode of the neutral (ν_2_ = 0.02 eV).^17,55^ Furthermore, the electron action spectrum (Figure 3) and the direct detachment portions of the photoelectron spectra (red, Figure 1) are dominated by excitation of different vibrational progressions (ν_2_ vs ν_1_) indicating that the respective final states i.e. IO_2_^–^(^1^B_1_) and IO_2_(^2^B_1_), have different geometries (both are accessed from the same initial state IO_2_^–^(^1^A_1_)). Therefore, on balance, it is most likely that we are observing a vibrational progression arising from a bound, valence electronically excited anion state, as previously observed for IO^–^ and CuF_2_^–^.^4,56^

The observation of multiple autodetachment features in some photoelectron spectra, indicates that autodetachment from a vibrational level of the ^1^B_1_ state can access a number of final states, i.e. internally excited IO_2_. Effectively, after population of a single autodetaching resonance, the final state distribution is determined by Franck–Condon factors arising from the overlap of the populated vibrational level of the IO_2_^–^(^1^B_1_) state with the final IO_2_(^2^B_1_) state. The energy spacing between the autodetachment peaks (eKE ∼ 0.01 eV, 0.04 eV, 0.07 eV and 0.09 eV, blue, Figure 1) is similar to the vibrational frequency of the bending mode of IO_2_ (ν_2_ = 0.02 eV).^17^ Given that we may have evidence for excitation of the bending mode in IO_2_^–^(^1^B_1_), autodetachment leading to IO_2_ with excitation in the bending mode seems reasonable.

The autodetaching vibrational levels of the ^1^B_1_ state are of shape resonance character because of the one electron transition involved ((b_1_)^1^(a_1_)^1^ → (b_1_)^1^(a_1_)^0^), suggesting that autodetachment is expected to be fast. Furthermore, given the autodetaching electron is of s-character (loss of (a_1_) electron), there is no centrifugal barrier, consistent with the observation of very low energy autodetached electrons (blue, Figure 1). By considering the width of the peaks in the action spectra (fwhm, Δ*E* ∼ 0.015 eV), we can extract a lifetime of $\Delta t\geq \frac{\hbar }{2\Delta E}$ ∼ 20 fs, which while prone to error from experimental factors broadening the peak width, is consistent with a short-lived excited state. This is also consistent with a valence bound anion state, as dipole bound states often have longer lifetimes (∼ps).^57,58^

As the *hν* is increased, the number of autodetachment features in the photoelectron spectra increases, as the range of final states which are energetically accessible increases. Additionally, the relative intensities of the autodetachment features change compared with each other, as well as direct detachment, indicating that the propensity for each transition (photoexcitation/autodetachment/direct detachment) changes with *hν*. The changing relative intensity of autodetachment features (blue) compared to direct detachment features (red) in the photoelectron spectra (Figure 1) likely arises from the competition between photoexcitation and photodetachment, which have different cross sections, selection rules and Franck–Condon envelopes, such that the propensity for both processes shifts independently with *hν*. Similar, and often complex, changes in the relative preference for direct detachment and autodetachment have been seen for other anions due to the location and nature of anionic resonances.^56,59–65^ Furthermore, though there seems to be a clear preference for the eKE ∼ 0.01 eV feature (blue, Figure 1) to be the largest autodetachment feature, suggesting a preference for maximizing the internal energy in IO_2_ following autodetachment, the relative intensity of the other autodetachment features does change. This may arise as a result of the changing overlap of the anion resonance wave function, with the detachment continuum, as the vibrational excitation in the IO_2_^–^(^1^B_1_) state changes. Alternatively, the dynamics on the ^1^B_1_ state following photoexcitation may affect the final state distribution following autodetachment. For example, other anions with large dipole moments have shown interconversion between dipole bound and valence bound anion states before electron loss.^66^ To investigate the excited state dynamics of IO_2_^–^ further, we would like to perform time-resolved photoelectron spectroscopy in the future, but it is beyond the scope of the current work. A cartoon depicting the likely autodetachment pathways is shown in Figure 4.

**Figure 4:**
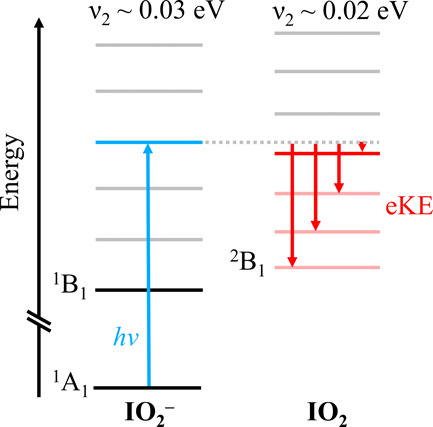
Cartoon depicting the photoexcitation (*hν*) of a single anion resonance (blue), followed by autodetachment to a range of final vibrational states of IO_2_, where dark red indicates the most likely final state (maximum internal energy in IO_2_) and pale red indicates observed, but less favored final states. The red arrows indicate the eKE of electrons lost via autodetachment.

As IO_2_^–^ is observed in the atmosphere, evidence for a bound electronically excited state is of significant importance. The vertical excitation energies for the bound state, and vibrationally excited anion resonances, are all in the visible region, where the solar spectrum has significant intensity. Therefore, it is probable that photoexcitation of IO_2_^–^ is occurring in the atmosphere, leading to an electronically excited IO_2_^–^ or to electron loss and the formation of IO_2_. Furthermore, our results indicate that IO_2_ is preferentially formed in an internally excited state, likely with excitation of the bending mode, which may enhance/change the chemistry it can subsequently undergo in the atmosphere. Additionally, although we cannot directly interrogate any other photoproducts using our current methods, it is possible that this excited state mediates further photochemistry which we cannot observe e.g. dissociation. Ultimately, the potential implications are that the photochemistry of IO_2_^–^ may play a significant, and to date unconsidered, role in the chemistry of the atmosphere through the formation of electrons, internally excited IO_2_, and, potentially, further highly reactive iodine-containing ions and radicals.

## Data Availability

The data, which support the findings reported here, are available online at https://doi.org/10.5281/zenodo.20346011.
